# Large-Area WS_2_ Film with Big Single Domains Grown by Chemical Vapor Deposition

**DOI:** 10.1186/s11671-017-2329-9

**Published:** 2017-10-03

**Authors:** Pengyu Liu, Tao Luo, Jie Xing, Hong Xu, Huiying Hao, Hao Liu, Jingjing Dong

**Affiliations:** School of Science, China University of Geosciences, Beijing, 100083 China

**Keywords:** Two-Dimensional Materials, Tungsten Disulfide, Chemical Vapor Deposition (CVD), Transition Metal Dichalcogenides (TMDCs)

## Abstract

High-quality WS_2_ film with the single domain size up to 400 μm was grown on Si/SiO_2_ wafer by atmospheric pressure chemical vapor deposition. The effects of some important fabrication parameters on the controlled growth of WS_2_ film have been investigated in detail, including the choice of precursors, tube pressure, growing temperature, holding time, the amount of sulfur powder, and gas flow rate. By optimizing the growth conditions at one atmospheric pressure, we obtained tungsten disulfide single domains with an average size over 100 μm. Raman spectra, atomic force microscopy, and transmission electron microscopy provided direct evidence that the WS_2_ film had an atomic layer thickness and a single-domain hexagonal structure with a high crystal quality. And the photoluminescence spectra indicated that the tungsten disulfide films showed an evident layer-number-dependent fluorescence efficiency, depending on their energy band structure. Our study provides an important experimental basis for large-area, controllable preparation of atom-thick tungsten disulfide thin film and can also expedite the development of scalable high-performance optoelectronic devices based on WS_2_ film.

**Electronic supplementary material:**

The online version of this article (10.1186/s11671-017-2329-9) contains supplementary material, which is available to authorized users.

## Background

Two-dimensional materials have important research significance in the future electronics and optoelectronics because of their unique planar advantages, quantum confinement effect, and lack of interlayer interference. As a typical two-dimensional material, graphene possesses many outstanding properties, such as super high carrier mobility, excellent thermal conductivity, outstanding flexibility, and ultrafast photoresponse, which have presented as a promising material in a wide range of application fields of next-generation flexible electronics, optoelectronics, and energy storage [1]. Massless Dirac fermions endow graphene with ultrahigh carrier mobility, but its semi-metallic property with zero bandgap greatly limits its use in devices. In recent years, the graphene-like monolayer transition metal dichalcogenides have aroused widespread interest in the scientific community and become a research focus in semiconductor micro- and nanoelectronics due to its moderate bandgap, excellent carrier mobility, and tunable electrical and optical properties.

Two-dimensional transition metal dichalcogenides (2D TMDCs) usually have a generalized chemical formula as MX_2_, where M is a transition metal of groups 4–10 (Mo, W, etc.) and X is a chalcogen (S, Se, Te, etc.). MX_2_ is a typical layered compound, every unit cell of which contains three layers of atoms (X-M-X). The intralayer atoms are tightly bound with covalent bonds, and the interlayer atoms are coupled by weak van der Waals force [2]. Similar to MoS_2_, monolayer WS_2_ has many novel physical properties distinguished from its bulk, such as direct bandgap, strong spin-orbit coupling, and intense interaction between light and matter. Therefore, it has promising potential application in future optoelectronic and micro/nanoelectronic devices [3]. So far, several routes of fabricating single-layer two-dimensional materials have been established, such as mechanical exfoliation, film sulfurization, thermal decomposition, and chemical vapor deposition. Among them, mechanical exfoliation suffers from the drawbacks of low yield, poor repeatability, and limited size [4]. In film sulfurization, a thin W or WO_3_ film is firstly sputtered on the substrate, and then, the W/WO_3_ film is sulfurized in sulfur-vapor atmosphere. The process is relatively simple, but the thickness of the film is difficult to control [5–7]. Liu et al. synthesized MoS_2_ films by thermal decomposition. After being soaked in (NH_4_)_6_MoS_4_ solution, the substrate was taken out and heated at 500 °C in an Ar/H_2_ atmosphere. Finally the WS_2_ film with a large area and a uniform thickness was obtained; however, the crystalline quality of the films was poor [8]. Chemical vapor deposition (CVD) has become an important and widely used technique for growing two-dimensional TMDC materials due to its easy operation, good controllability, relatively mature fabrication routes, and high yield of large-area few-layer film [9–13].

Since 2011, many research groups in the world successfully have synthesized atom-thick WS_2_ films by the CVD method. Zhang et al. synthesized the atomic-layered triangular WS_2_ film on sapphire substrate by low-pressure chemical vapor deposition with single domain size up to 50 μm [14]. Cong et al. improved the CVD method by putting a single-end-sealed smaller-diameter quartz tube inside a bigger-diameter quartz tube and sandwiching a trace of WO_3_ powder between two wafers. This method effectively increases the precursor concentration and pressure in the tube, and the obtained single-domain size was up to 178 μm [3]. Considering that the WO_3_ precursor has a high sublimation temperature, Li et al. introduce a proper amount of alkali metal halides in chemical vapor deposition reaction as growth promoters. Alkali metal halide (MX, M=Na or K, X=Cl, Br or I) could reduce the reaction temperature by about 100 °C through forming volatile tungsten oxyhalide species, which facilitated the delivery of the precursor to the growth substrate. However, the addition of alkali halide in reaction tube inevitably introduces impurities and pollutes the reaction products [15]. Both Yanfeng Zhang’s and Kyung Nam Kang’s group reported that adding an appropriate concentration of H_2_ contributes to the rapid sublimation and sulfurization of WO_3_ precursor, because the reducibility of H_2_ is stronger than that of S [14, 16]. Fu et al. studied the effects of gas flow rate and reaction temperature on the morphology and domain size of WS_2_ films in an argon-hydrogen mixture (97%Ar + 3%H_2_) atmosphere. They got 52 μm WS_2_ flakes by optimizing the CVD growth conditions [17]. Rong et al. used a two-temperature-zone furnace to precisely control sulfur introduction time to achieve an ideal large-area WS_2_ film growth with single domain size up to 370 μm [18]. Although CVD method has many advantages, it is still urgent and very challenging to coordinate the intricate and complicated relationship among many growth parameters. At present, arising from the high sublimate temperature of WO_3_ precursor and the potential danger in using Ar and H_2_ gas mixtures during growth, the preparation of high-quality WS_2_ films with large domain size still faces great challenges. In this work, we did a systematic and deep study on the growth rules of WS_2_ films synthesized by CVD technique. For the first time, we investigated comprehensively the impact of different growing parameters on the morphology of WS_2_ films, such as precursor types, gas pressure, growing temperature, holding time, the amount of sulfur powder, gas flow rate, and substrate position. By optimizing the processing conditions, large-area WS_2_ films with big single domains were obtained via an atmospheric-pressure chemical vapor deposition (AP-CVD) method. The films were examined by Raman, atomic force microscopy (AFM), transmission electron microscopy (TEM), and photoluminescence (PL) measurements to have an excellent crystalline quality. Our study paves a way to fabricate large monolayer WS_2_ single crystal with excellent properties, which is critical to building scalable devices.

## Methods

**Fig. 1:**
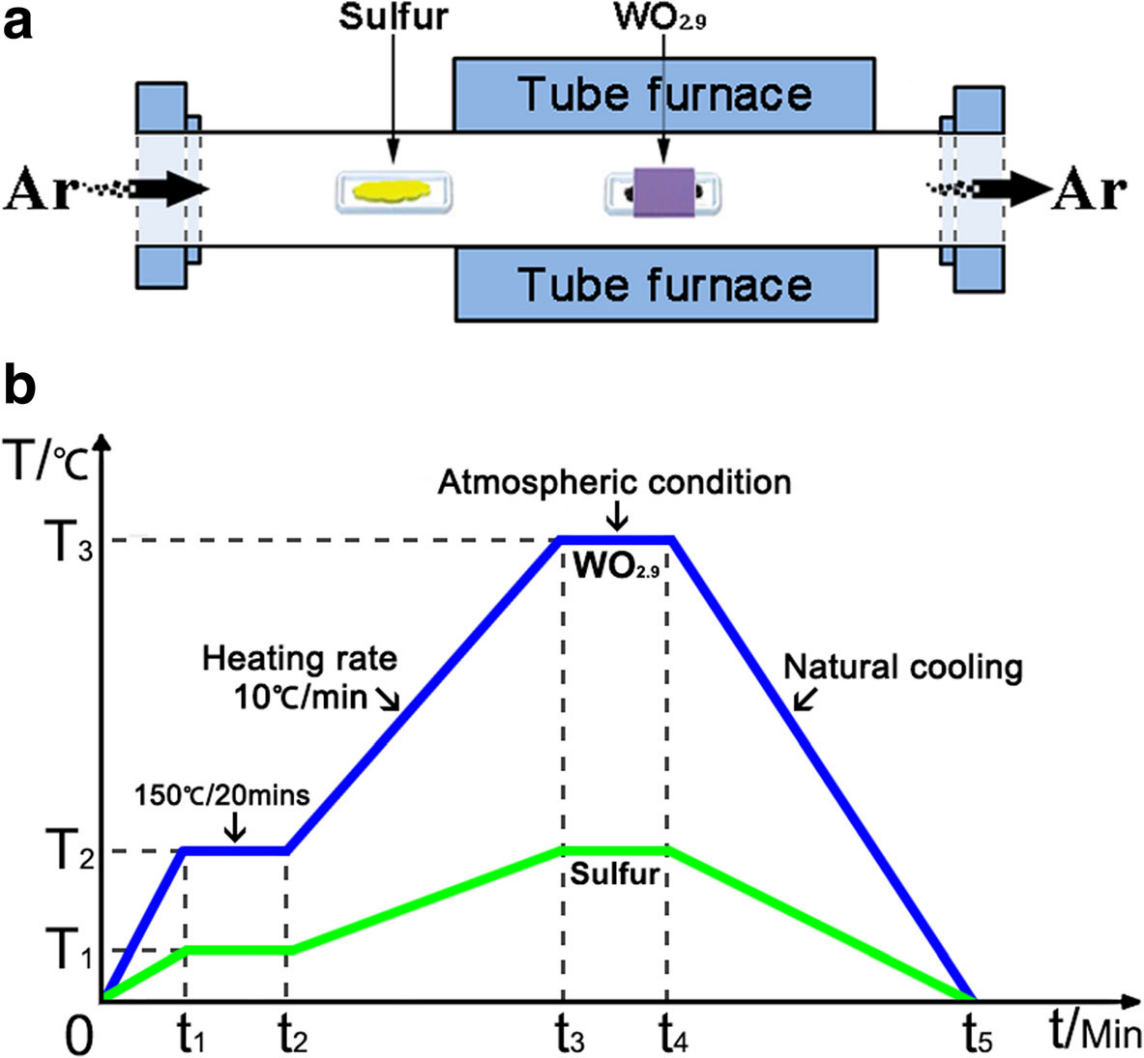
**a** The schematic setup of CVD furnace. **b** The heating and cooling curves of WO_2.9_ and S powder

To synthesize WS_2_ film, WO_3_ (Sigma-Aldrich, 99.9%), WO_2.9_ (Alfa Aesar, 99.99%) and S (Alfa Aesar, 99.0%) powders were used as W and S precursors, respectively. In a typical growth process, a single-side-polished SiO_2_/Si wafer was firstly cleaned in ethanol, isopropanol, and deionized water in sequence by ultrasonic cleaning for 15 min. A small amount of tungsten oxide powder (0.1 g) was uniformly spread on the bottom of a crucible, and the Si wafer with a thickness of 300 nm SiO_2_ was put upside down with the polished side facing towards the tungsten oxide powder. Then, the crucible was located at the center of quartz tube (60 mm in diameter) as shown in Fig. 1a. The quartz boat with sulfur powder inside was placed in the upstream region of the quartz tube and warmed up by the radiation heat of the tube furnace. After the quartz tube was pumped down to a pressure of 100 mTorr, the quartz tube was purged using Ar gas at 500 sccm for 30 min, and then, the Ar gas was controlled at a constant flow rate until the reaction was finished. The furnace was heated firstly from room temperature to 150 °C and dwelled at this temperature for 20 min to remove moisture in the tube. Then, the temperature continued to increase to the desired value with a heating rate of 10 °C/min. After reaching the growth temperature, the furnace kept the temperature for a period of time. At the end of the growth process, the quartz tube was cooled down to room temperature naturally. To be clear, the whole temperature control scheme is shown in Fig. 1b.

The morphologies of as-grown WS_2_ flakes were characterized by a Hitachi S4800 scanning electron microscope with an acceleration voltage of 5–10 kV. Raman measurements were conducted using Nanophoton Raman-11 microscope with an ultra-high speed imaging capability. The Si peak at 520 cm^−1^ was used as a reference for wavenumber calibration. Steady-state PL spectra were taken by a confocal micro-PL system. A 532-nm excitation laser was focused on the sample by using a large numerical aperture objective with a spot size around 1–2 μm in diameter. Topography images of the sample were obtained by using atomic force microscope (Bruker multimode 8) in tapping mode. A field emission JEOL JEM-2100F was operated at 200 kV for high-resolution TEM (HRTEM) and selected-area electron diffraction (SAED) imaging.

## Results and Discussion

In CVD synthesis, the growth of two-dimensional TMDCs is impacted by many factors, such as pressure, temperature, gas flow rate, and growing time. These factors are very important in the growth of high-quality and large-area 2D WS_2_ film. In this paper, the influence of each of these factors on the morphology of WS_2_ films is firstly discussed in detail, and then, the optimum growth conditions for large-area few-layer films are determined. Finally, in order to examine the films’ structure and crystal quality, the characterization results under the optimized experimental conditions are presented, including Raman, AFM, PL, and TEM.

### WO_3_ and WO_2.9_

**Fig. 2:**
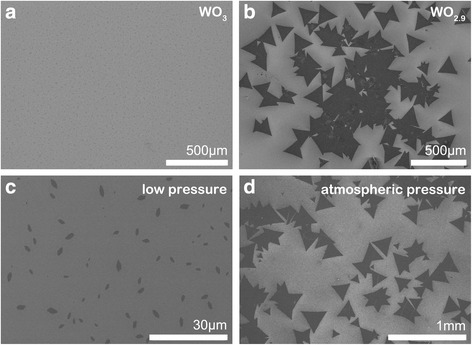
**a**, **b** The SEM images of WS_2_ films prepared using WO_3_ and WO_2.9_ as W source, respectively. **c**, **d** The SEM images of WS_2_ samples prepared at low pressure and one atmospheric pressure, respectively

We used WO_3_ and WO_2.9_ powder as two distinct precursors to investigate their effects on the growth of WS_2_ film. Fig. 2a, b shows SEM images of WS_2_ films grown with precursors of WO_3_ and WO_2.9_, respectively. When WO_3_ was used as W source, it was hardly to see WS_2_ film on the substrate, which was further confirmed by Raman measurements. However, when WO_2.9_ was used as precursor, there appeared a large number of triangular WS_2_ domains on the substrate. After dozens of repeated experiments, we found that the yield of triangular WS_2_ with WO_2.9_ precursor was much higher than that in the case of WO_3_ as precursor. The reproducibility of this experiment result was over 90%. van der Vlies et al. have studied the basic reaction steps in the sulfidation of WO_3_ crystal [19]. They found that W^6+^ cannot be directly sulfurized by S unless some intermediates are formed due to the high W–O bond energy. The reduction of W^6+^ to W^5+^ is mandatory for an incorporation of sulfur in the WO_3_ lattice. For WO_2.9_ in our case, its partial W^6+^ ions have been reduced to W^5+^ or W^4+^ ions. Therefore, we think the substitution of W^5+^ or W^4+^ for W^6+^ at the initial stage facilitates the growth of the single crystal WS_2_ film.

### Tube Pressure

For this study, we adopted two pressure values during the growth of WS_2_ film in experiments: low pressure (< 100 mTorr) and one atmospheric pressure (1 atm). Figure 2c shows a SEM image of the sample prepared under a low-pressure environment. As we found, only leaf-like flakes were randomly distributed over the substrate, which were not WS_2_ but WO_3_, as was further confirmed by Raman characterization. Figure 2d shows a SEM image of the sample grown under one atmospheric pressure. In contrast with Fig. 2c, there were many triangular domains with size above 100 μm appearing on the substrate, which were WS_2_ as evidenced by Raman spectroscopy. Such contrast experiments have been repeated many times, and every time, we got almost the same results. Compared with low-pressure mode, atmospheric pressure mode was more helpful to get a high yield of WS_2_ flakes with large size and clear edges. As we know, CVD process generally includes two stages: (1) gas transportation and gas phase reaction and (2) surface adsorption and surface reaction. In both these two stages, collision process is a very important and robust factor. At one atmospheric pressure, the mean free path of gas molecules becomes shorter and the collision frequency gets higher (see Additional file 1). The higher collision frequency combined with high temperature and high flow rate usually lead to a higher reaction rate and a higher nucleation rate by promoting the chemical reaction between precursors or between precursor clusters and substrate. On the other hand, from the theory of thermodynamics, the chemical free-energy change ΔG (< 0) is the driving force for nucleation. The critical free-energy change ΔG^*^ could be regarded as an energy barrier of nucleation, which is inversely correlated with the gas pressure [20]. Therefore, the higher pressure in APCVD always leads to a smaller nucleation energy barrier, a higher nucleation rate, and a larger nucleation density than those in the case of low pressure CVD (LPCVD). So, the atmospheric pressure mode in our experiments is more favored in growing 2D TMDC films.

### Growth Temperature

**Fig. 3:**
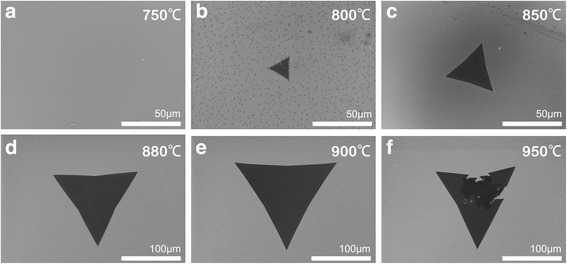
The SEM images of WS_2_ samples prepared at 750 °C (**a**), 800 °C (**b**), 850 °C (**c**), 880 °C (**d**), 900 °C (**e**), and 950 °C (**f**), respectively

Based on the above results, we chose WO_2.9_ as W source and adjusted the tube pressure as one atmospheric pressure. In the following, we investigate the influence of growth temperature on the crystalline quality of WS_2_ film. We conducted a series of experiments by varying the furnace temperature at 750, 800, 850, 880, 900, and 950 °C, respectively. As shown in Fig. 3, with increasing temperature, the average domain size of WS_2_ films first increased and then decreased. Low temperature induced low diffusion rate of the precursor, so that the precursor can be readily trapped at pre-growth sites on the substrate. At the very early precursor nucleation stage, most nucleation sites formed trap centers and the subsequent precursor nucleated at those trapping sites (Fig. 3b). As a result, many spot-like WS_2_ domains were obtained. With raising temperature, the formation of new phases became more difficult due to the increased critical nucleus radius and the boosted nucleation free energy barrier, which restrained nucleation and deposition of WS_2_ on the substrate, resulting in a decreased nucleation density. At the same time, the molecular thermal kinetic energy increased significantly, which facilitated the surface diffusion of WO_3 − *x*_ and the reaction of WO_3 − *x*_ with S. Thus, it is in line with better crystallization of WS_2_ lattices and increased flake size (Fig. 3c–e). However, when the growth temperature was further raised to 950 °C, the overall flake did not grow larger but slightly smaller, and some flakes exhibited some cracks during growth as shown in Fig. 3f. We conjecture the cracks may happen at the sites of grain boundaries or defects, where the chemical bond is relatively fragile and easy to be broken up by high temperature.

### Holding Time

**Fig. 4:**
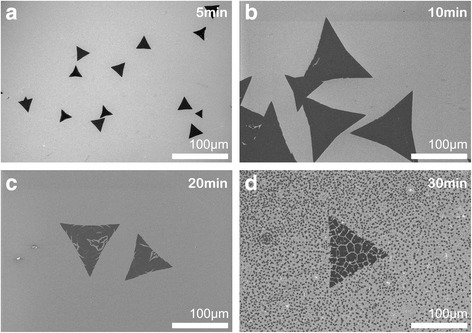
The SEM images of WS_2_ films prepared at 5 min (**a**), 10 min (**b**), 20 min (**c**), and 30 min (**d**), respectively

In this section, the holding time in our experiments was controlled at 5, 10, 20, and 30 min, respectively. The growth temperature was set as 900 °C, and the amount of S powder was fixed at 0.7 g. As shown in Fig. 4, with the increase of the holding time, the domain size of the films expanded continuously, from about 30 μm at 5 min to about 120 μm at 10 min. However, the lateral size did not continue to enlarge further for the holding time of 20 min or even 30 min. We speculate that it might be related with multiple factors, for example, the substrate surface roughness, nucleation density, and gas molecular diffusion rate. From the thermodynamics theory, the change of free energy during growing may also determine or even limit its lateral size. Additionally, the existing complete triangular film would inevitably suffer from frequent impingement from surrounding gas molecules, which may pollute or even destroy the original film, causing defects in the films. These defects may keep spreading at high temperature and finally damage the original complete film, as shown in Fig. 4d.

### The Amount of the Sulfur Powder

**Fig. 5:**
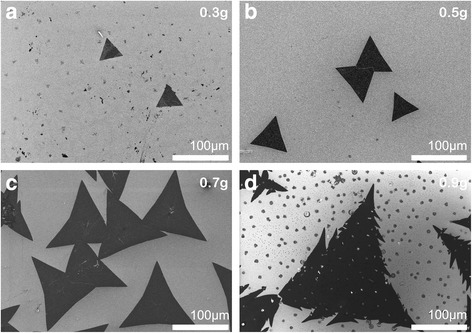
The SEM images of WS_2_ films grown under different sulfur quantities: 0.3 g (**a**), 0.5 g (**b**), 0.7 g (**c**), and 0.9 g (**d**), respectively

The amount of the sulfur powder used in growth is also a very important factor. Although the growth dynamics of two-dimensional materials are not fully understood, yet, it is generally accepted that two possible growth modes dominate in the 2D TMDC material growth: (1) MO_3 − *x*_ species adsorb and diffuse on the surface of the substrate as well as react with sulfur vapor atoms to form MS_2_; (2) MO_3 − *x*_ groups react directly with S atoms in gas phase, and the resulting MO_*x*_S_*y*_ clusters adsorb, nucleate, and grow on the substrate. Clearly, these two modes should be in direct competition depending on the sulfur concentration in the environment. In our experiments, we loaded the amount of sulfur powder as 0.3, 0.5, 0.7, and 0.9 g, respectively, in order to study the influence of the S vapor concentration on the growth of WS_2_ film. When the sulfur powder was only 0.3 g, the partial pressure of sulfur vapor in quartz tube was relatively low, which easily produced incompletely sulfurized film. As shown in Fig. 5a, in addition to few triangular WS_2_ flakes with size around 30 μm, there were many small irregular-shaped spots (edge size < 5 μm). These small spots were checked by Raman spectroscopy and proved not to be WS_2_. With the sulfur powder addition from 0.5 to 0.7 g, a large number of triangle-shaped WS_2_ domains appeared on the substrate, and their average size increased from ~ 50 μm to over 100 μm (Fig. 5b, c). When the sulfur powder was further added up to 0.9 g, larger domains with ~ 300 μm edge length and many small particles were present on the substrate, as shown in Fig. 5d. Analyzed by energy-dispersive spectroscopy (EDS) measurement (see Additional file 1), most of these small particles were WO_*x*_S_*y*_ grains, acting as seed nuclei and constantly reacting with S atoms to form WS_2_ flakes [21]. The adjacent flakes with the same crystalline orientation grew up with time and finally merged into a larger domain. It is easy to see in Fig. 5d that the large triangular domain was obviously made up of many smaller triangular domains. Under high sulfur partial pressure, the number of crystal nuclei increased significantly. Adjacent nuclei grew competitively, and at the same time, their crystal orientations became disordered, resulting in a rough edge. Feldman et al. claimed that the high S partial pressure can lead to WO_3 − *x*_ nanoparticles wrapped up by a layer of WS_2_ inorganic fullerene structure [22, 23], which would suppress the further reaction between the WO_3 − *x*_ core and the outer sulfur atoms. So, we could see some particles remaining on the film surface. It should be noted that with the adding of the sulfur powder, the edges of triangular-like flakes became more concave, which is possibly caused by the markedly different growth rate of the S zigzag (S-zz) edges and the W zigzag (W-zz) edges [24]. In sum, the experimental results presented here reveal that loading a proper amount of sulfur powder plays a critical role in the growth of high-quality two-dimensional WS_2_ films.

### Gas Flow Rate

**Fig. 6:**
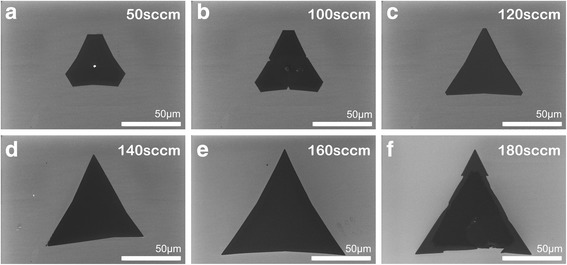
The SEM images of WS_2_ films prepared at different Ar gas flow rates: 50 sccm (**a**), 100 sccm (**b**), 120 sccm (**c**), 140 sccm (**d**), 160 sccm (**e**), and 180 sccm (**f**), respectively

In this part, we set gas flow rates as 50, 100, 120, 140, 160, and 180 sccm, respectively, to explore the influence of gas flow rate on film growth. Other growth conditions were regulated as the aforementioned optimized parameter. As shown in Fig. 6, when the flow rate of Ar increased from 50 to 180 sccm, WS_2_ domains experienced a morphology transformation as well as a size change. For the gas flow rate of 50 sccm, the WS_2_ film was dominated by ~ 40 μm truncated triangular domains. As the flow rate increased from 50 to 120 sccm, the truncated side became shorter and shorter, and finally, the flakes turned into a triangular shape with sharp and smooth edges. At the same time, the average domain size was obviously enlarged to ~ 60 μm. Then, the domains stop to change their shape and continued to grow to ~ 100 μm at a flow rate of 160 sccm. When the gas flow rate reached 180 sccm, no further increase in size was observed. As we know, WS_2_ bulk has a trigonal prismatic crystal structure (2H phase), where each W atom is prismatically coordinated to six surrounding S atoms, forming a thermodynamically stable phase. We assume that all shapes of domains start growing from a hexagonal nucleus with three sides of W zigzag (W-zz) terminations and another three sides of S zigzag (S-zz) terminations. As the gas flow rate increased, more S vapor atoms were brought to the center of the quartz tube and induced a smaller Mo to S atomic concentration ratio. Warner et al. investigated the influence of Mo/S atom ratio on the morphology of MoS_2_ films based on the principle of crystal growth [24]. According to their work, when the W:S atom ratio gradually changed to less than 1:2, three W-zz terminations grew faster than another three S-zz terminations, which would result in the domain shape transformation from hexagon into truncated triangle and finally into equilateral triangle. Also, the high flow rate promoted the mass transfer process, which contributed to the increase in the crystal growth rate. As the gas flow rate was further raised from 160 to 180 sccm, instability may occur as atoms did not have enough time to move to the right lattice locations, and the probability of defect formation and anisotropy of growth increased owing to the local thermal disturbance and the local imbalance of precursor concentration and pressure. Therefore, at high gas flow rate, the smooth edges of WS_2_ films are easy to get rough as shown in Fig. 6f.

### The Position of Substrate

**Fig. 7:**
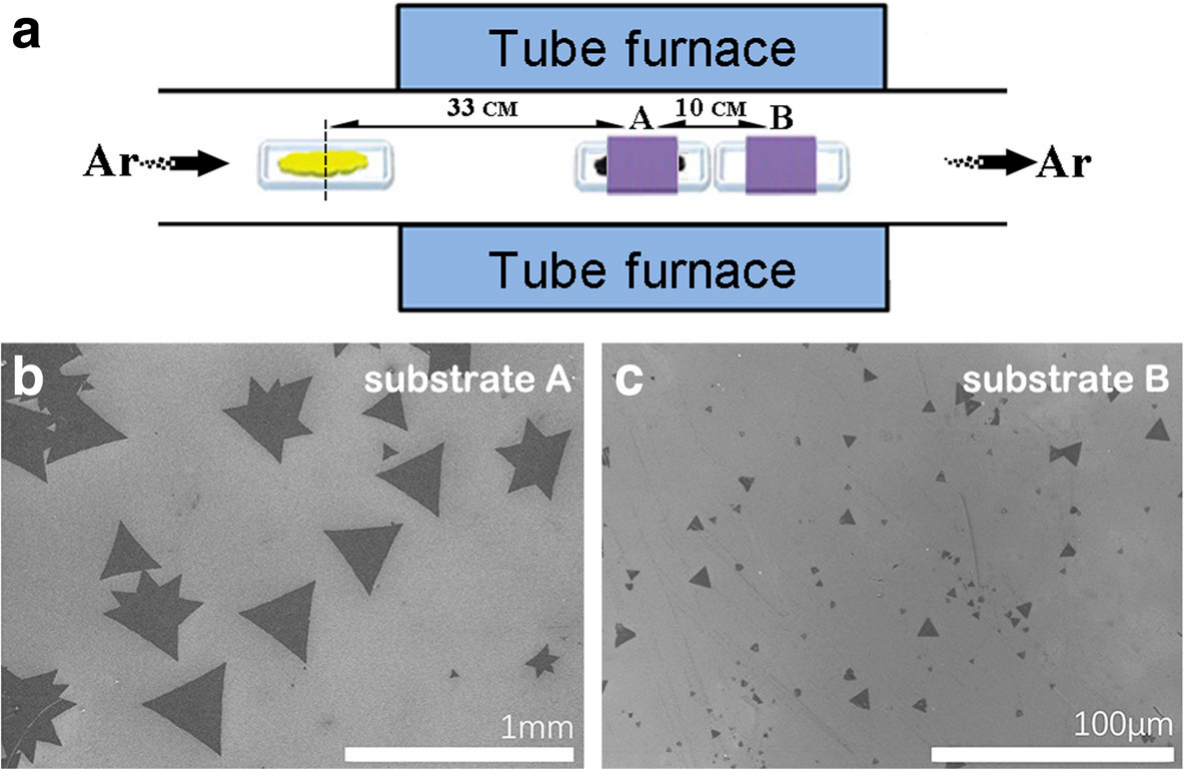
**a** The schematic experimental setup with the position of substrates A and B. **b**, **c** The SEM images of synthesized WS_2_ films on substrates A and B, respectively

Last but not the least, the position of substrate was also a key parameter to WS_2_ growth. Here, we make a comparison between two substrate positions. Substrate A was placed above the alumina boat and faced down the tungsten oxide powder, and substrate B was positioned at the downstream as shown in Fig. 7a. At the position of the tube center, the higher temperature determined a higher supersaturated concentration of the precursor, which always led to a smaller crystallization nucleus density. At the same time, due to sufficient precursor supply and high atomic diffusion rate, it was easier to grow large-area WS_2_ single domains (~ 200 μm) on substrate A. In contrast, for substrate B placed at the downstream, the lower temperature resulted in a reduced supersaturation of the precursor, which easily brought about more nuclei appearing on the substrate. Meanwhile, owing to the low precursor concentration and low molecular kinetic energy, the single domains on substrate B (~ 10 μm) grow much smaller than those on substrate A, as is shown in Fig. 7b, c.

### Optimization and Characterization

**Fig. 8:**
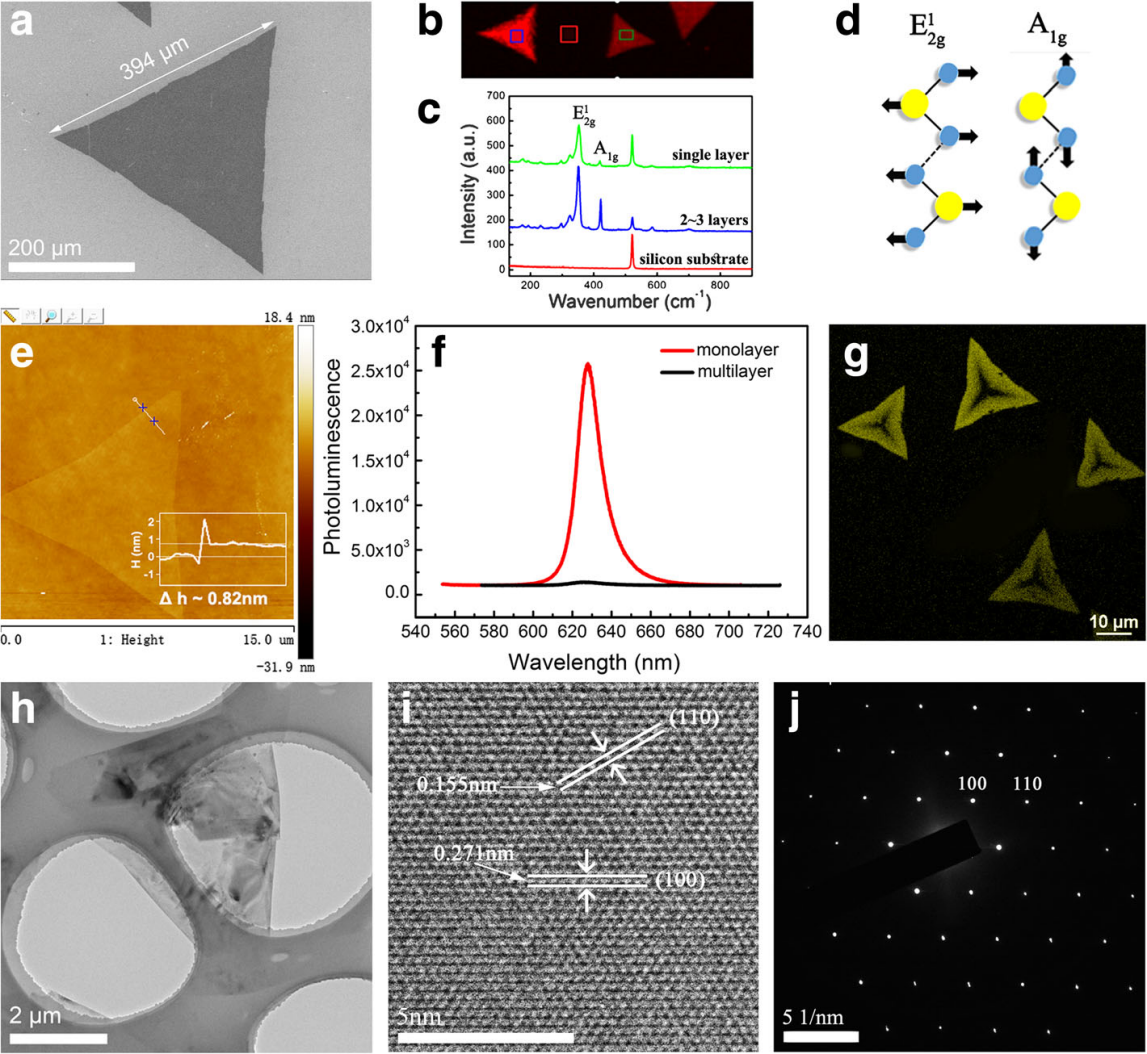
**a** The SEM image of synthesized WS_2_ film. **b**, **c** Raman mapping and the corresponding Raman spectra of WS_2_ flakes. **d** The schematic of two typical Raman vibration modes E^1^_2g_ and A_1g_. **e** An AFM image and height profile for a WS_2_ single domain. **f** The PL spectra of a monolayer and a multilayer WS_2_ flake. **g** Fluorescent image of monolayer WS_2_ flakes. **h** TEM image of a WS_2_ flake on a copper grid. **i**, **j** High-resolution TEM image and its corresponding SAED pattern of the freely suspended monolayer WS_2_ on a TEM grid

In preceding work, a series of experiments have been carried out to investigate the impacts of the growth parameters on the morphology evolution of WS_2_ film, including growing temperature, holding time, gas flow rate, and the quantity of sulfur. Our results enable us to realize controllable monolayer WS_2_ growth and also provide some general guidelines for other 2D material growth. Based on the above experiment results, we obtained the optimal experimental conditions for the growth of high-quality large-area WS_2_ film: 0.1 g WO_2.9_ and 0.7 g S powder are taken as W and S precursors, respectively; the substrate is located right above the alumina boat facing down the WO_2.9_ powder; the growth temperature is controlled at 900 °C and kept for 10 min; the Ar gas flow rate is set as 160 sccm with tube pressure maintaining at one atmospheric pressure. Figure 8a shows a SEM image of a typical WS_2_ single domain synthesized under the optimized condition. The domain has a complete and regular triangular shape with sharp and smooth edge length of ~ 400 μm, which is much bigger than the edge size of WS_2_ domain prepared by micromechanical exfoliation.

Raman spectroscopy has been widely used to study 2D materials, from which the information of molecular vibration and rotation in the material can be extracted. So, it offers a fingerprint spectrum to be used to identify the structure of the material. Figure 8b exhibits a typical Raman mapping of a WS_2_ film constructed by plotting A_1g_ mode intensity, which clearly shows a perfect triangular shape. Figure 8c shows Raman spectra of the three different areas marked by colored boxes in Fig. 8b over a frequency range of 100–900 cm^−1^. The measurement was performed at room temperature with 532 nm laser excitation. In addition to the Raman peak at 520 cm^−1^ from substrate Si, the two distinct peaks at ~ 352.5 and ~ 419 cm^−1^ denote typical WS_2_ optical phonon vibration modes E^1^_2g_ and A_1g_. These two modes correspond to the in-plane and out-of-plane vibrations of WS_2_ lattice, respectively, which are shown in Fig. 8d. With decreasing film thickness, the A_1g_ peak is redshifted, and concurrently, the E^1^_2g_ mode is blueshifted due to the weakened interlayer interaction, leading to a decrease in the frequency separation between the two modes. Therefore, the frequency separation is often used to identify the thickness of the two-dimensional material. For the left WS_2_ single domain labeled with blue box (Fig. 8b), the Raman frequency difference of the E^1^_2g_ and A_1g_ mode is around 71 cm^−1^, where the two peaks’ intensity ratio (A_1g_/E^1^_2g_) is about 0.5, as shown in Fig. 8c. The high intensity of the A_1g_ peak confirms the two- to three-layered structure of the crystal. For the right WS_2_ flake labeled with green box, the Raman frequency interval reduces to 67 cm^−1^, and the intensity ratio of the two peaks is about 0.21. At the same time, the significant reduction in the intensity of A_1g_ peak than that of the E^1^_2g_ peak confirms a monolayer WS_2_ [25].

Atomic force microscopy (AFM) is an effective tool to measure the surface topography of materials by “touching” the sample surface with a mechanical probe. The information of the WS_2_ film thickness can be obtained directly by AFM measurement. A height image of a WS_2_ single domain and the line profile across the flake clearly show a height of 0.82 nm (Fig. 8e), which is in the height range of a single-layer WS_2_ film and consistent with the results reported in the literatures [10, 14].

To study the details of light emission from the CVD WS_2_ flakes, micro-photoluminescence (m-PL) spectroscopy measurement and PL intensity mapping were performed (with 532 nm laser excitation). As shown in Fig. 8f, the PL intensity of monolayer WS_2_ is much stronger than that of multilayer. It is well known that the electronic band structure transitions from indirect to direct bandgap as WS_2_ is thinned down from multilayer to monolayer. Strong emission is observed only for the monolayer. Furthermore, the strong PL peak located at 627 nm is in agreement with the reported direct bandgap of ~ 2 eV [26, 27]. The full width at half maximum (FWHM) value of ~ 47 meV is close to those from mechanically exfoliated monolayers in previous reports [28, 29]. Figure 8g shows the PL intensity image of the triangular WS_2_ monolayer, which exhibits non-uniform emission intensity across the flakes. The edges emit the brightest light, and the strength of the emission gradually decays when moving towards the body center and eventually becomes invisible. Similar results have been reported in other papers [3, 26]. Cong et al. explained the suppressing of PL at the center might be due to the existence of structural and charge defects. For instance, S vacancies are inevitably induced in CVD growth of WS_2_ films. The related lattice defects and dislocations could become the non-irradiative recombination centers for excitons, which could result in heavily reduced PL emission intensity.

Finally, we utilized TEM and SAED to evaluate the crystallinity of WS_2_ flakes. Figure 8h gives a typical low-magnification TEM image of a triangular WS_2_ flake on a holy carbon-coated copper grid. The flake was broken during transfer process, but we still can clearly see that the surface of the film is clean, free from other contaminants. The HRTEM image (Fig. 8i) reveals the hexagonal ring lattice consisting of alternating tungsten atoms and sulfur atoms. The corresponding SAED pattern further confirmed its hexagonal symmetry. The first-order diffraction spots, corresponding to (100) planes, were used to calculate the interspacing *d* of (100) planes. We found that *d* (100) equals to 0.271 nm, which is in agreement with the results deduced from HRTEM measurement. Also, the interspacing *d* (110) is deduced to be 0.155 nm according to the (110) diffraction spots in SAED pattern. Both interplanar distances coincide well with those of bulk WS_2_ [14].

## Conclusions

We systematically investigated the influence of various synthesis parameters on the morphology evolution of WS_2_ film grown by chemical vapor deposition, such as precursors, pressure, growth temperature, holding time, amount of sulfur powder, gas flow rate, and source-substrate distance. Based on the optimized experimental conditions, large-area WS_2_ thin films with single domain size up to ~ 400 μm have been successfully prepared on Si/SiO_2_ wafer. The crystal structure, layer number, and luminescence of the WS_2_ films have been examined by Raman spectra, transmission electron microscopy, atomic force microscopy, and photoluminescence. We believe our results will lead to further progress in improving the crystalline quality and large-area growth of the exciting 2D transitional metal dichalcogenides (TMDCs). At the same time, this work will push forward the applications of TMDC film in the fields of micro-(nano-) optoelectronics, photovoltaic industry, photocatalysis, and energy storage.

## Additional file

Additional file 1: Supplementary material for WS_2_ film. **Figure S1.** SEM pictures of WS_2_ flakes synthesized from different batches. **Figure S2.** SEM pictures of WS_2_ films before (a) and after (b) being annealed at 950 °C under Ar atmosphere. (DOCX 2790 kb)

## Abbreviations

2D: Two-dimensional
AFM: Atomic force microscopy
APCVD: Atmospheric pressure chemical vapor deposition
EDS: Energy-dispersive spectroscopy
FWHM: Full width at half maximum
LPCVD: Low pressure chemical vapor deposition
PL: Photoluminescence
SAED: Selected-area electron diffraction
SEM: Scanning electron microscopy
TEM: Transmission electron microscopy
TMDCs: Transition metal dichalcogenides
