# Supplementary material for: Large-Area WS2 Film with Big Single Domains Grown by Chemical Vapor Deposition
Source: Nanoscale Res Lett. 2017 Oct 3;12:558. doi: 10.1186/s11671-017-2329-9 (PMC5626679; doi:10.1186/s11671-017-2329-9)
Supplement: Supplementary file 1 — Supplementary material for WS2 film. Figure S1. SEM pictures of WS2 flakes synthesized from different batches. Figure S2. SEM pictures of WS2 films before (a) and after (b) being annealed at 950 °C under Ar atmosphere. (DOCX 2790 kb) [file 11671_2017_2329_MOESM1_ESM.docx]

Supplementary Material

**Large-area WS_2_ film with big single domains grown by chemical vapor deposition**

Pengyu Liu, Tao Luo, Jie Xing^[[1]](#footnote-1)^*, Hong Xu, Huiying Hao, Hao Liu, Jingjing Dong

**Note 1**

Under optimized conditions, it is often seen that the WS_2_ flakes with domain size of ~400 μm are present in our samples. Fig. S1 shows some WS_2_ flakes with big domain size synthesized from different batches.


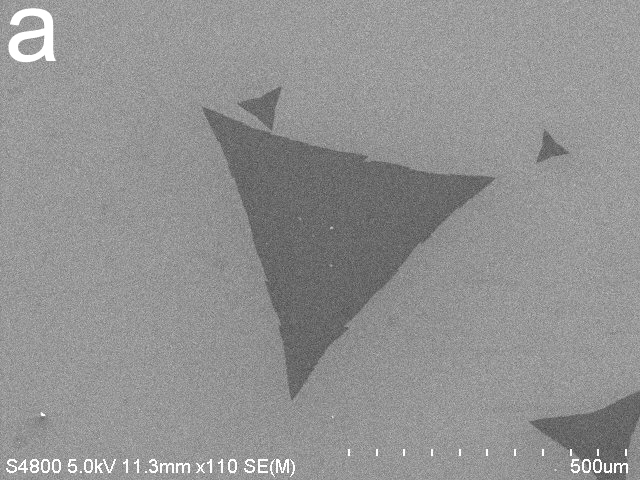

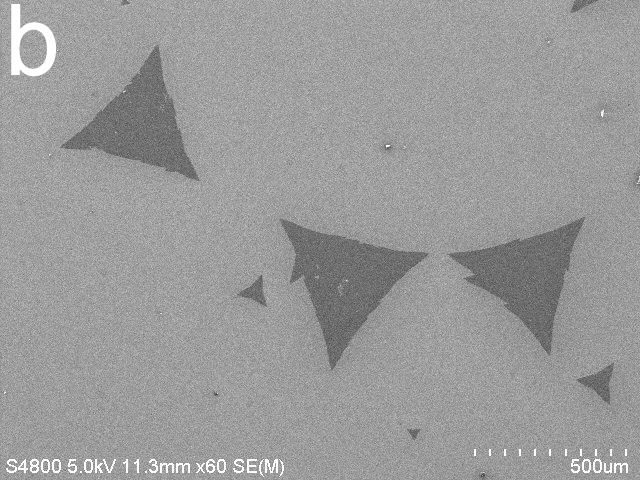

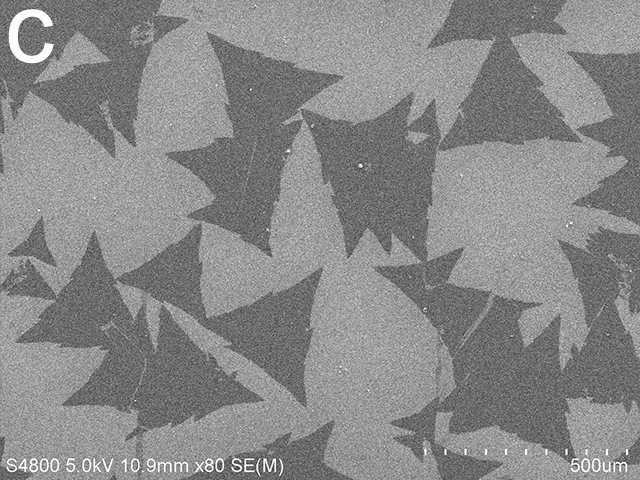


Fig. S1 SEM pictures of WS_2_ flakes synthesized from different batches

**Note 2**

In order to check the thermal stability of WS_2_ film at 950 ^o^C, we annealed pieces of WS_2_ sample with complete and well-shaped triangular morphology in tube furnace and heated up to 950 ^o^C for 10 mins under pure Ar environment. SEM images taken before and after the annealing are shown in Fig. S2. It is clear almost all the WS_2_ films disappeared after the annealing process and only some vague triangular outlines remained, which illustrates the instability of WS_2_ film at such high temperature when with no external supply of precursors.


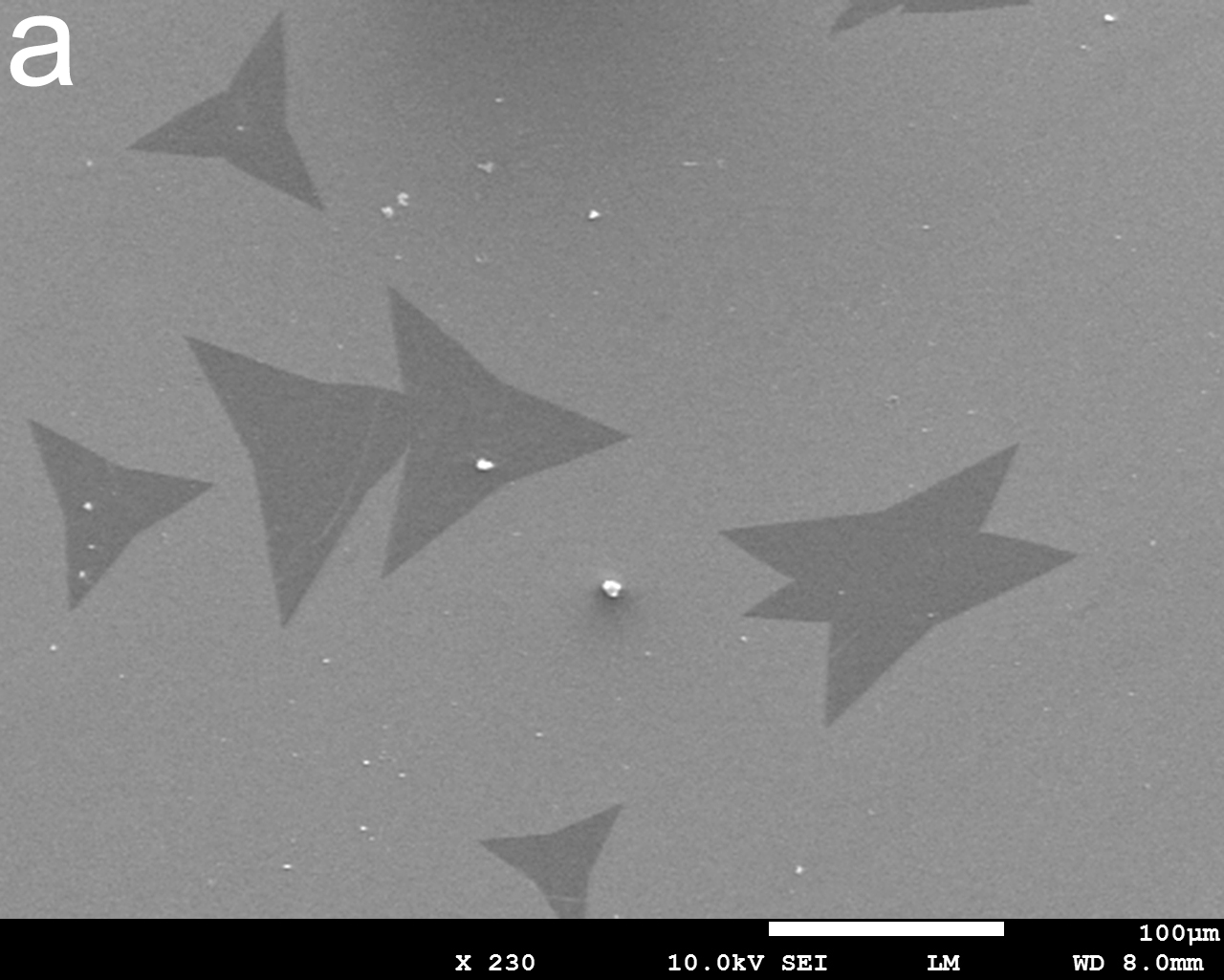

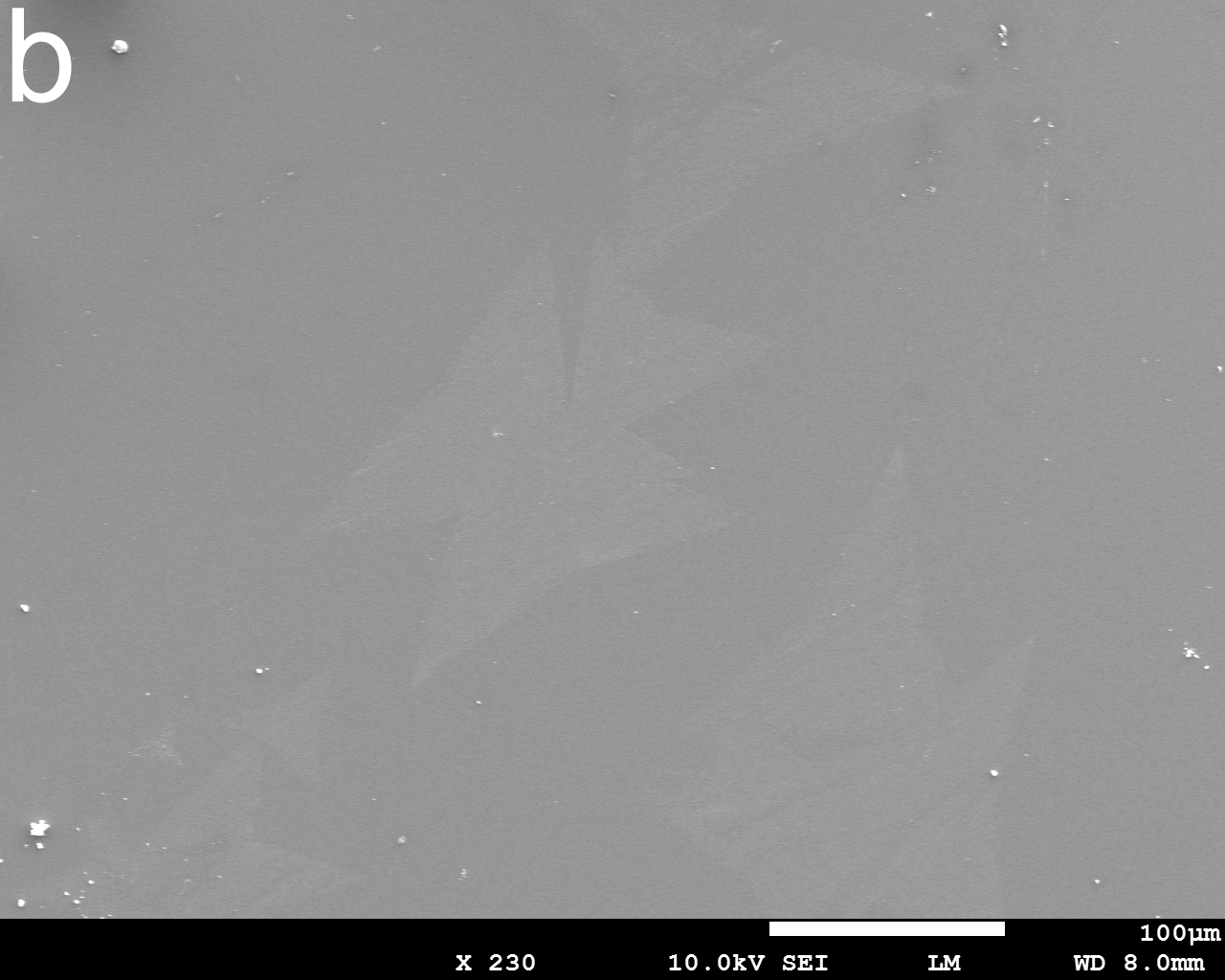


Fig. S2 SEM pictures of WS_2_ films before (a) and after (b) being annealed at 950 ^o^C under Ar atmosphere

**Note 3**

Based on the formulae $=\frac{\mathrm{kT}}{\sqrt{2}\pi d^{2}P}$ and $z=\sqrt{2}\pi d^{2}\bar{v}\frac{P}{\mathrm{kT}}$ , where k, P, T denote the Boltzmann constant, pressure, and temperature, respectively, the mean free path λ of gas molecules becomes shorter and the collision frequency z gets higher with increasing the pressure in the quartz tube.

**Note 4**

In order to examine the composition of the particles on some WS_2_ films, we performed EDS measurement. The composition analysis is listed in the following pages. It is clearly seen that most of particles contain W, O and S elements. The Si element comes from the substrate (SiO_2_/Si) due to the longer penetration depth of electron beam. And the C element comes from the conductive tape. The EDS measurement on these particles further confirmed its WO_x_S_y_ nature.


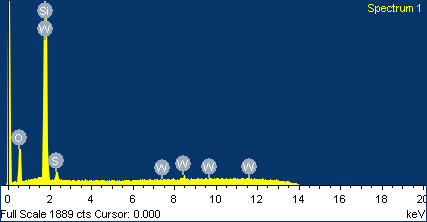

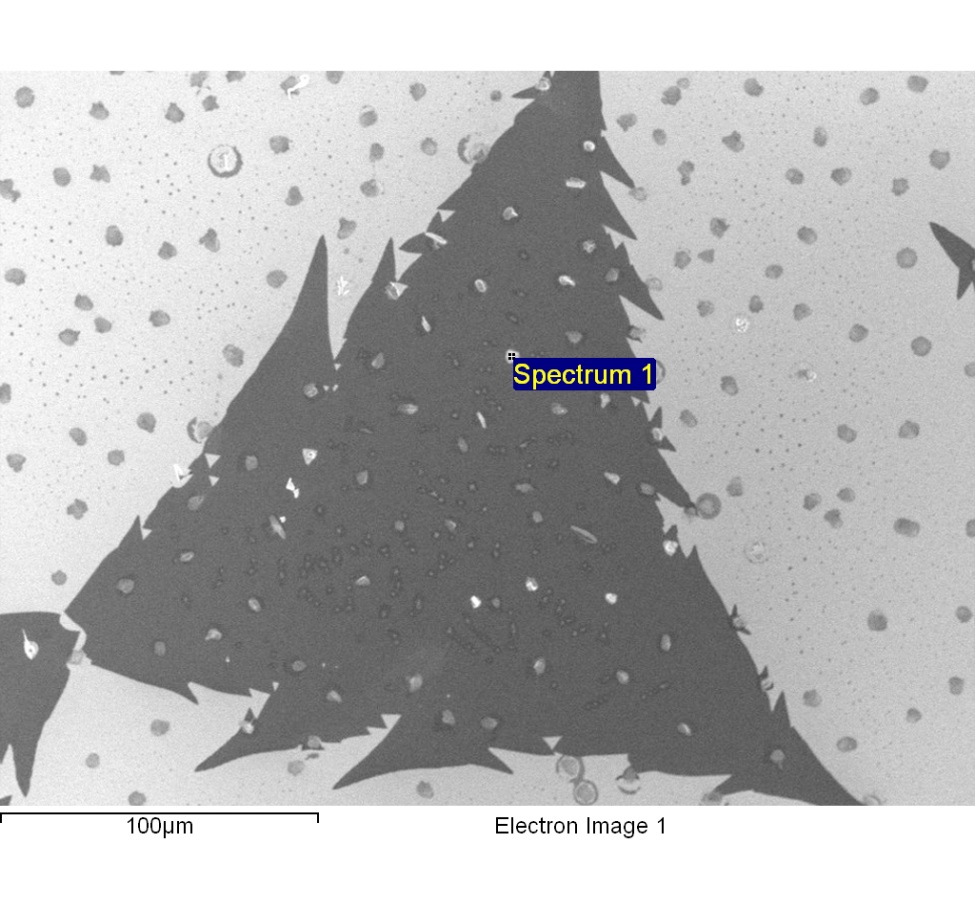

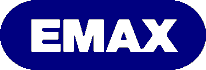

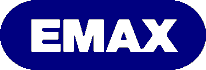

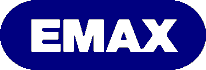

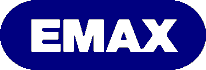

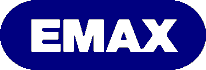

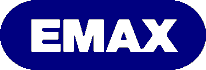


谱图处理 :

没有被忽略的峰

处理选项 : 所有经过分析的元素 (已归一化)

重复次数 = 3

标准样品 :

C CaCO3 1-Jun-1999 12:00 AM

O SiO2 1-Jun-1999 12:00 AM

Si SiO2 1-Jun-1999 12:00 AM

S FeS2 1-Jun-1999 12:00 AM

| 元素 | 重量 | 原子 |  |
| --- | --- | --- | --- |
|  | 百分比 | 百分比 |  |
| C K | 5.93 | 11.44 |  |
| O K | 17.90 | 25.91 |  |
| Si K | 74.67 | 61.57 |  |
| S K | 1.50 | 1.08 |  |
|  |  |  |  |
| 总量 | 100.00 |  |  |

注释：

2017-8-31 19:29:44

20151220

谱图处理 :

没有被忽略的峰

处理选项 : 所有经过分析的元素 (已归一化)

重复次数 = 3

标准样品 :

O SiO2 1-Jun-1999 12:00 AM

Si SiO2 1-Jun-1999 12:00 AM

S FeS2 1-Jun-1999 12:00 AM

Ca Wollastonite 1-Jun-1999 12:00 AM

W W 1-Jun-1999 12:00 AM

Re Re 1-Jun-1999 12:00 AM

| 元素 | 重量 | 原子 |  |
| --- | --- | --- | --- |
|  | 百分比 | 百分比 |  |
| O K | 19.19 | 57.22 |  |
| Si K | 7.38 | 12.54 |  |
| S K | 0.51 | 0.76 |  |
| Ca K | 11.36 | 13.52 |  |
| W M | 58.19 | 15.10 |  |
| Re M | 3.37 | 0.86 |  |
|  |  |  |  |
| 总量 | 100.00 |  |  |

注释：

2017-8-31 19:33:34

项目 1

谱图处理 :

没有被忽略的峰

处理选项 : 所有经过分析的元素 (已归一化)

重复次数 = 3

标准样品 :

O SiO2 1-Jun-1999 12:00 AM

Na Albite 1-Jun-1999 12:00 AM

W W 1-Jun-1999 12:00 AM

| 元素 | 重量 | 原子 |  |
| --- | --- | --- | --- |
|  | 百分比 | 百分比 |  |
| O K | 16.57 | 60.91 |  |
| Na K | 5.54 | 14.16 |  |
| W M | 77.90 | 24.92 |  |
|  |  |  |  |
| 总量 | 100.00 |  |  |

注释：

2017-8-31 19:42:23

20150913-1

谱图处理 :

没有被忽略的峰

处理选项 : 所有经过分析的元素 (已归一化)

重复次数 = 3

标准样品 :

O SiO2 1-Jun-1999 12:00 AM

S FeS2 1-Jun-1999 12:00 AM

Ca Wollastonite 1-Jun-1999 12:00 AM

W W 1-Jun-1999 12:00 AM

| 元素 | 重量 | 原子 |  |
| --- | --- | --- | --- |
|  | 百分比 | 百分比 |  |
| O K | 11.41 | 45.79 |  |
| S K | 5.76 | 11.54 |  |
| Ca K | 10.99 | 17.60 |  |
| W M | 71.83 | 25.08 |  |
|  |  |  |  |
| 总量 | 100.00 |  |  |

注释：

2017-8-31 19:46:00

项目 1

谱图处理 :

可能被忽略的峰 : 0.250, 3.335 keV

处理选项 : 所有经过分析的元素 (已归一化)

重复次数 = 3

标准样品 :

O SiO2 1-Jun-1999 12:00 AM

Na Albite 1-Jun-1999 12:00 AM

S FeS2 1-Jun-1999 12:00 AM

W W 1-Jun-1999 12:00 AM

| 元素 | 重量 | 原子 |  |
| --- | --- | --- | --- |
|  | 百分比 | 百分比 |  |
| O K | 19.29 | 65.78 |  |
| Na K | 3.11 | 7.38 |  |
| S K | 2.71 | 4.62 |  |
| W M | 74.89 | 22.22 |  |
|  |  |  |  |
| 总量 | 100.00 |  |  |

注释：

2017-8-31 19:50:32

项目 1

Spectrum processing :

No peaks omitted

Processing option : All elements analyzed (Normalised)

Number of iterations = 3

Standard :

O SiO2 1-Jun-1999 12:00 AM

Si SiO2 1-Jun-1999 12:00 AM

S FeS2 1-Jun-1999 12:00 AM

W W 1-Jun-1999 12:00 AM

| Element | Weight% | Atomic% |  |
| --- | --- | --- | --- |
|  |  |  |  |
| O K | 24.07 | 38.99 |  |
| Si K | 61.47 | 56.73 |  |
| S K | 3.35 | 2.71 |  |
| W M | 11.12 | 1.57 |  |
|  |  |  |  |
| Totals | 100.00 |  |  |

Comment:

S1


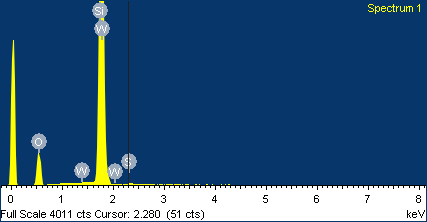

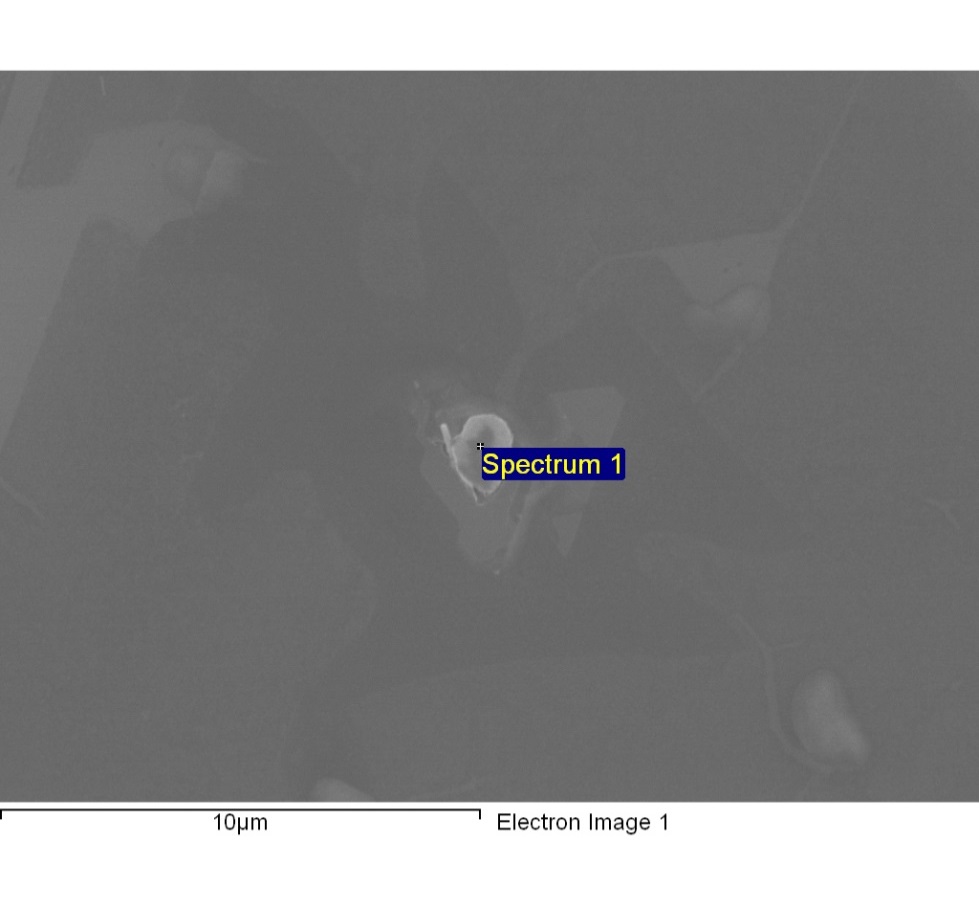

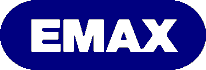

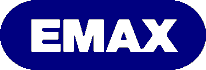

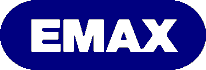

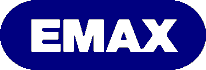

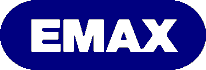

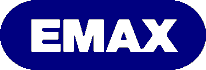


谱图处理 :

没有被忽略的峰

处理选项 : 所有经过分析的元素 (已归一化)

重复次数 = 3

标准样品 :

C CaCO3 1-Jun-1999 12:00 AM

O SiO2 1-Jun-1999 12:00 AM

Si SiO2 1-Jun-1999 12:00 AM

S FeS2 1-Jun-1999 12:00 AM

| 元素 | 重量 | 原子 |  |
| --- | --- | --- | --- |
|  | 百分比 | 百分比 |  |
| C K | 5.93 | 11.44 |  |
| O K | 17.90 | 25.91 |  |
| Si K | 74.67 | 61.57 |  |
| S K | 1.50 | 1.08 |  |
|  |  |  |  |
| 总量 | 100.00 |  |  |

注释：

2017-8-31 19:29:44

20151220

谱图处理 :

没有被忽略的峰

处理选项 : 所有经过分析的元素 (已归一化)

重复次数 = 3

标准样品 :

O SiO2 1-Jun-1999 12:00 AM

Si SiO2 1-Jun-1999 12:00 AM

S FeS2 1-Jun-1999 12:00 AM

Ca Wollastonite 1-Jun-1999 12:00 AM

W W 1-Jun-1999 12:00 AM

Re Re 1-Jun-1999 12:00 AM

| 元素 | 重量 | 原子 |  |
| --- | --- | --- | --- |
|  | 百分比 | 百分比 |  |
| O K | 19.19 | 57.22 |  |
| Si K | 7.38 | 12.54 |  |
| S K | 0.51 | 0.76 |  |
| Ca K | 11.36 | 13.52 |  |
| W M | 58.19 | 15.10 |  |
| Re M | 3.37 | 0.86 |  |
|  |  |  |  |
| 总量 | 100.00 |  |  |

注释：

2017-8-31 19:33:34

项目 1

谱图处理 :

没有被忽略的峰

处理选项 : 所有经过分析的元素 (已归一化)

重复次数 = 3

标准样品 :

O SiO2 1-Jun-1999 12:00 AM

Na Albite 1-Jun-1999 12:00 AM

W W 1-Jun-1999 12:00 AM

| 元素 | 重量 | 原子 |  |
| --- | --- | --- | --- |
|  | 百分比 | 百分比 |  |
| O K | 16.57 | 60.91 |  |
| Na K | 5.54 | 14.16 |  |
| W M | 77.90 | 24.92 |  |
|  |  |  |  |
| 总量 | 100.00 |  |  |

注释：

2017-8-31 19:42:23

20150913-1

谱图处理 :

没有被忽略的峰

处理选项 : 所有经过分析的元素 (已归一化)

重复次数 = 3

标准样品 :

O SiO2 1-Jun-1999 12:00 AM

S FeS2 1-Jun-1999 12:00 AM

Ca Wollastonite 1-Jun-1999 12:00 AM

W W 1-Jun-1999 12:00 AM

| 元素 | 重量 | 原子 |  |
| --- | --- | --- | --- |
|  | 百分比 | 百分比 |  |
| O K | 11.41 | 45.79 |  |
| S K | 5.76 | 11.54 |  |
| Ca K | 10.99 | 17.60 |  |
| W M | 71.83 | 25.08 |  |
|  |  |  |  |
| 总量 | 100.00 |  |  |

注释：

2017-8-31 19:46:00

项目 1

谱图处理 :

可能被忽略的峰 : 0.250, 3.335 keV

处理选项 : 所有经过分析的元素 (已归一化)

重复次数 = 3

标准样品 :

O SiO2 1-Jun-1999 12:00 AM

Na Albite 1-Jun-1999 12:00 AM

S FeS2 1-Jun-1999 12:00 AM

W W 1-Jun-1999 12:00 AM

| 元素 | 重量 | 原子 |  |
| --- | --- | --- | --- |
|  | 百分比 | 百分比 |  |
| O K | 19.29 | 65.78 |  |
| Na K | 3.11 | 7.38 |  |
| S K | 2.71 | 4.62 |  |
| W M | 74.89 | 22.22 |  |
|  |  |  |  |
| 总量 | 100.00 |  |  |

注释：

2017-8-31 19:50:32

项目 1

Spectrum processing :

No peaks omitted

Processing option : All elements analyzed (Normalised)

Number of iterations = 3

Standard :

O SiO2 1-Jun-1999 12:00 AM

Si SiO2 1-Jun-1999 12:00 AM

S FeS2 1-Jun-1999 12:00 AM

W W 1-Jun-1999 12:00 AM

| Element | Weight% | Atomic% |  |
| --- | --- | --- | --- |
|  |  |  |  |
| O K | 26.35 | 39.53 |  |
| Si K | 69.66 | 59.54 |  |
| S K | 0.66 | 0.50 |  |
| W M | 3.34 | 0.44 |  |
|  |  |  |  |
| Totals | 100.00 |  |  |

Comment:

S2


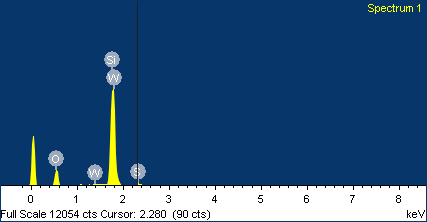

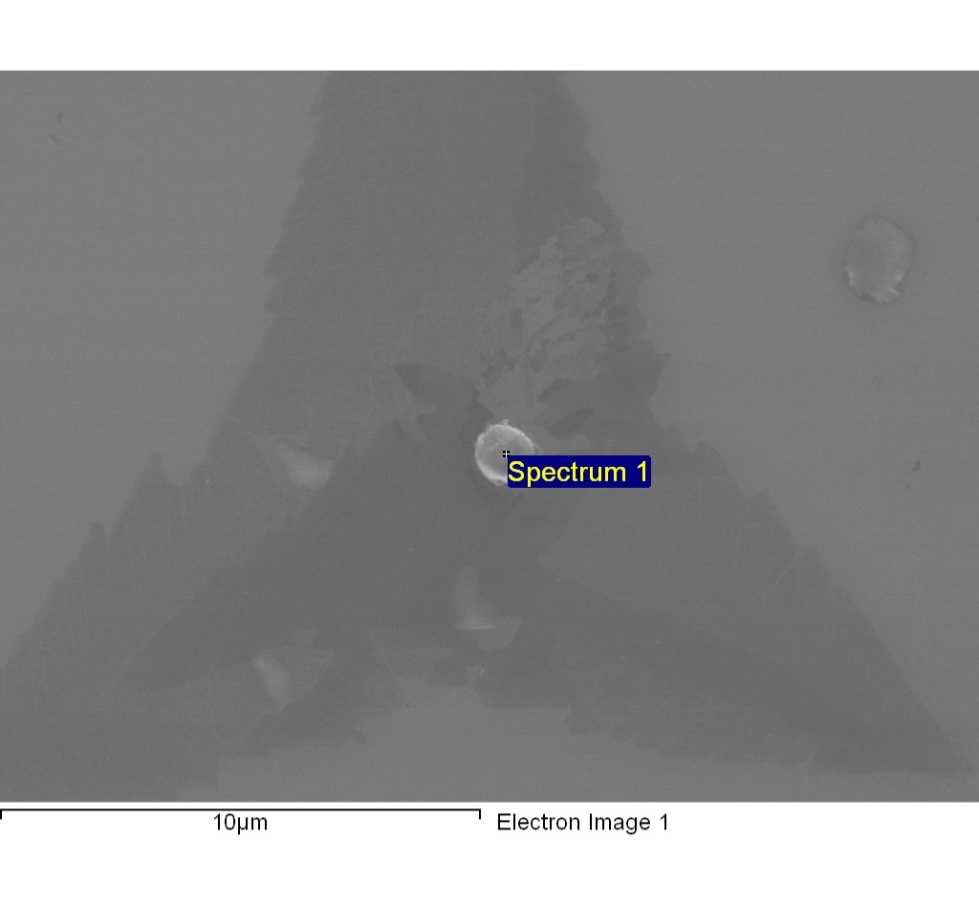

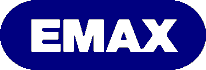

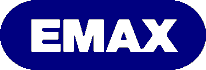

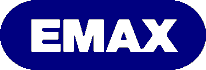


谱图处理 :

可能被忽略的峰 : 1.044 keV

处理选项 : 所有经过分析的元素 (已归一化)

重复次数 = 3

标准样品 :

O SiO2 1-Jun-1999 12:00 AM

Si SiO2 1-Jun-1999 12:00 AM

W W 1-Jun-1999 12:00 AM

| 元素 | 重量 | 原子 |  |
| --- | --- | --- | --- |
|  | 百分比 | 百分比 |  |
| O K | 18.00 | 29.68 |  |
| Si K | 73.55 | 69.10 |  |
| W M | 8.46 | 1.21 |  |
|  |  |  |  |
| 总量 | 100.00 |  |  |

注释：

2017-8-31 20:02:18

项目 1

谱图处理 :

可能被忽略的峰 : 0.250, 1.044 keV

处理选项 : 所有经过分析的元素 (已归一化)

重复次数 = 3

标准样品 :

O SiO2 1-Jun-1999 12:00 AM

Si SiO2 1-Jun-1999 12:00 AM

W W 1-Jun-1999 12:00 AM

| 元素 | 重量 | 原子 |  |
| --- | --- | --- | --- |
|  | 百分比 | 百分比 |  |
| O K | 22.17 | 36.37 |  |
| Si K | 66.32 | 61.99 |  |
| W M | 11.52 | 1.64 |  |
|  |  |  |  |
| 总量 | 100.00 |  |  |

注释：

2017-8-31 20:04:08

项目 1

Spectrum processing :

No peaks omitted

Processing option : All elements analyzed (Normalised)

Number of iterations = 3

Standard :

O SiO2 1-Jun-1999 12:00 AM

Si SiO2 1-Jun-1999 12:00 AM

S FeS2 1-Jun-1999 12:00 AM

W W 1-Jun-1999 12:00 AM

| Element | Weight% | Atomic% |  |
| --- | --- | --- | --- |
|  |  |  |  |
| O K | 32.26 | 48.93 |  |
| Si K | 56.08 | 48.46 |  |
| S K | 1.70 | 1.29 |  |
| W M | 9.96 | 1.31 |  |
|  |  |  |  |
| Totals | 100.00 |  |  |

Comment:

S3


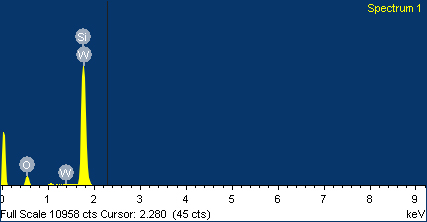

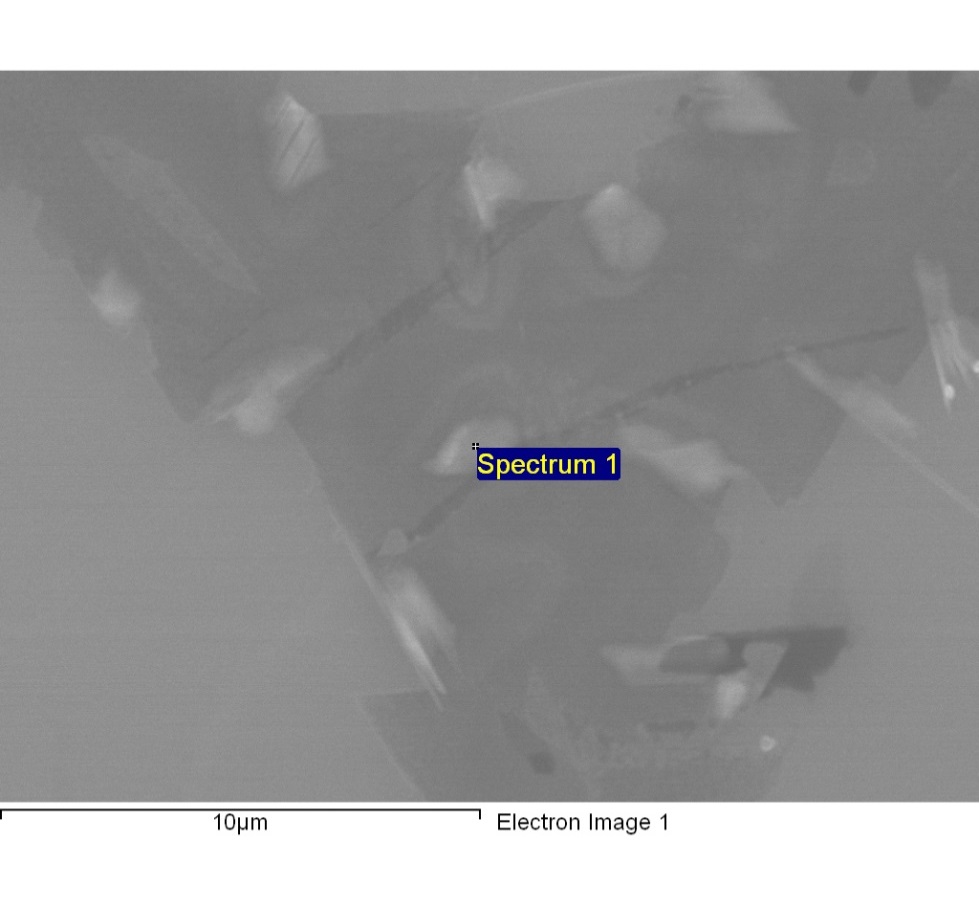

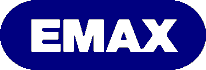


Spectrum processing :

Possible omitted peak : 1.042 keV

Processing option : All elements analyzed (Normalised)

Number of iterations = 3

Standard :

C CaCO3 1-Jun-1999 12:00 AM

O SiO2 1-Jun-1999 12:00 AM

Si SiO2 1-Jun-1999 12:00 AM

W W 1-Jun-1999 12:00 AM

| Element | Weight% | Atomic% |  |
| --- | --- | --- | --- |
|  |  |  |  |
| C K | 2.53 | 5.35 |  |
| O K | 21.13 | 33.56 |  |
| Si K | 65.90 | 59.64 |  |
| W M | 10.44 | 1.44 |  |
|  |  |  |  |
| Totals | 100.00 |  |  |

Comment:

S4


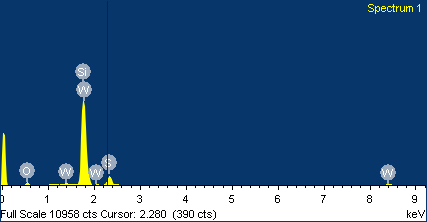

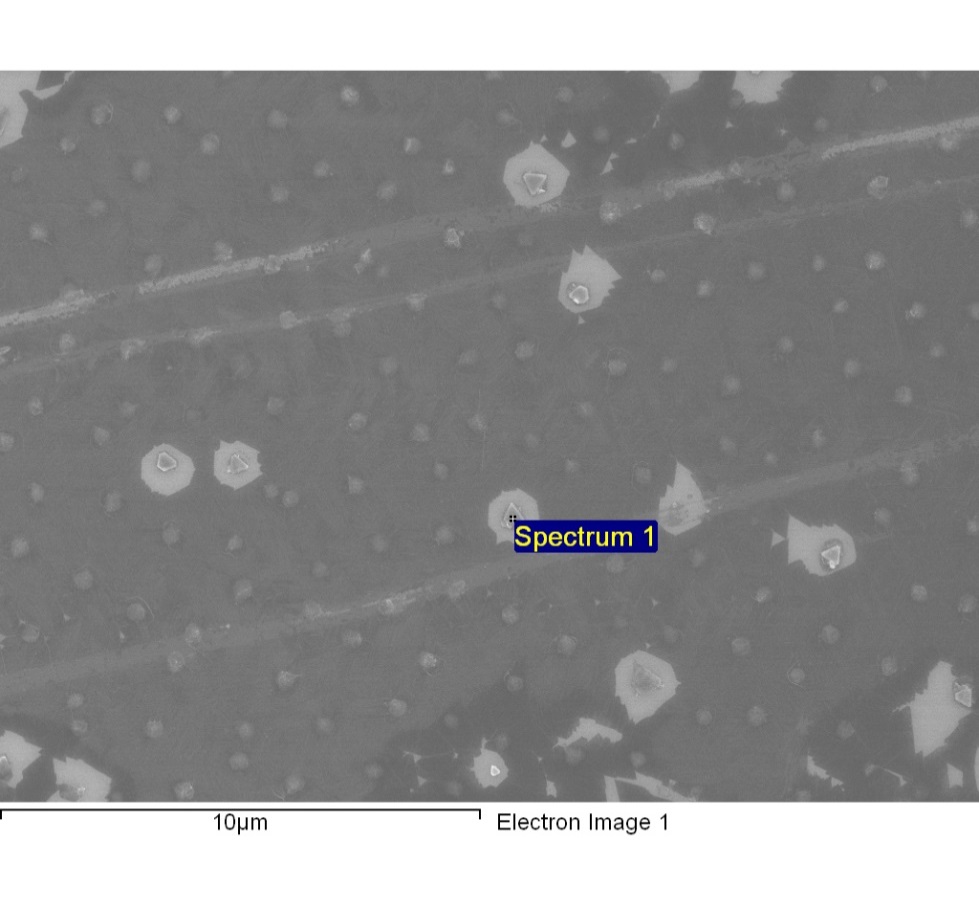

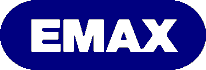

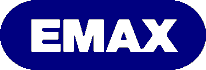

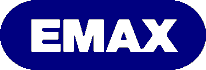

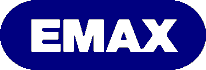

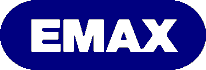


谱图处理 :

可能被忽略的峰 : 1.052 keV

处理选项 : 所有经过分析的元素 (已归一化)

重复次数 = 3

标准样品 :

O SiO2 1-Jun-1999 12:00 AM

Si SiO2 1-Jun-1999 12:00 AM

W W 1-Jun-1999 12:00 AM

| 元素 | 重量 | 原子 |  |
| --- | --- | --- | --- |
|  | 百分比 | 百分比 |  |
| O K | 19.29 | 30.84 |  |
| Si K | 75.09 | 68.38 |  |
| W M | 5.62 | 0.78 |  |
|  |  |  |  |
| 总量 | 100.00 |  |  |

注释：

2017-8-31 20:18:39

004

谱图处理 :

可能被忽略的峰 : 1.046 keV

处理选项 : 所有经过分析的元素 (已归一化)

重复次数 = 3

标准样品 :

O SiO2 1-Jun-1999 12:00 AM

Si SiO2 1-Jun-1999 12:00 AM

W W 1-Jun-1999 12:00 AM

| 元素 | 重量 | 原子 |  |
| --- | --- | --- | --- |
|  | 百分比 | 百分比 |  |
| O K | 20.78 | 33.66 |  |
| Si K | 70.60 | 65.13 |  |
| W M | 8.62 | 1.21 |  |
|  |  |  |  |
| 总量 | 100.00 |  |  |

注释：

2017-8-31 20:20:42

004

谱图处理 :

可能被忽略的峰 : 1.045 keV

处理选项 : 所有经过分析的元素 (已归一化)

重复次数 = 3

标准样品 :

O SiO2 1-Jun-1999 12:00 AM

Si SiO2 1-Jun-1999 12:00 AM

W W 1-Jun-1999 12:00 AM

| 元素 | 重量 | 原子 |  |
| --- | --- | --- | --- |
|  | 百分比 | 百分比 |  |
| O K | 21.97 | 38.20 |  |
| Si K | 59.56 | 59.00 |  |
| W M | 18.48 | 2.80 |  |
|  |  |  |  |
| 总量 | 100.00 |  |  |

注释：

2017-8-31 20:33:20

20151117-1-小-001

谱图处理 :

可能被忽略的峰 : 1.047 keV

处理选项 : 所有经过分析的元素 (已归一化)

重复次数 = 3

标准样品 :

O SiO2 1-Jun-1999 12:00 AM

Si SiO2 1-Jun-1999 12:00 AM

W W 1-Jun-1999 12:00 AM

| 元素 | 重量 | 原子 |  |
| --- | --- | --- | --- |
|  | 百分比 | 百分比 |  |
| O K | 20.59 | 36.80 |  |
| Si K | 58.97 | 60.02 |  |
| W M | 20.44 | 3.18 |  |
|  |  |  |  |
| 总量 | 100.00 |  |  |

注释：

2017-8-31 20:35:22

项目 1

Spectrum processing :

Possible omitted peak : 1.044 keV

Processing option : All elements analyzed (Normalised)

Number of iterations = 3

Standard :

C CaCO3 1-Jun-1999 12:00 AM

O SiO2 1-Jun-1999 12:00 AM

Si SiO2 1-Jun-1999 12:00 AM

S FeS2 1-Jun-1999 12:00 AM

W W 1-Jun-1999 12:00 AM

| Element | Weight% | Atomic% |  |
| --- | --- | --- | --- |
|  |  |  |  |
| C K | 3.19 | 8.77 |  |
| O K | 9.07 | 18.72 |  |
| Si K | 47.16 | 55.46 |  |
| S K | 11.47 | 11.81 |  |
| W M | 29.11 | 5.23 |  |
|  |  |  |  |
| Totals | 100.00 |  |  |

Comment:

S5


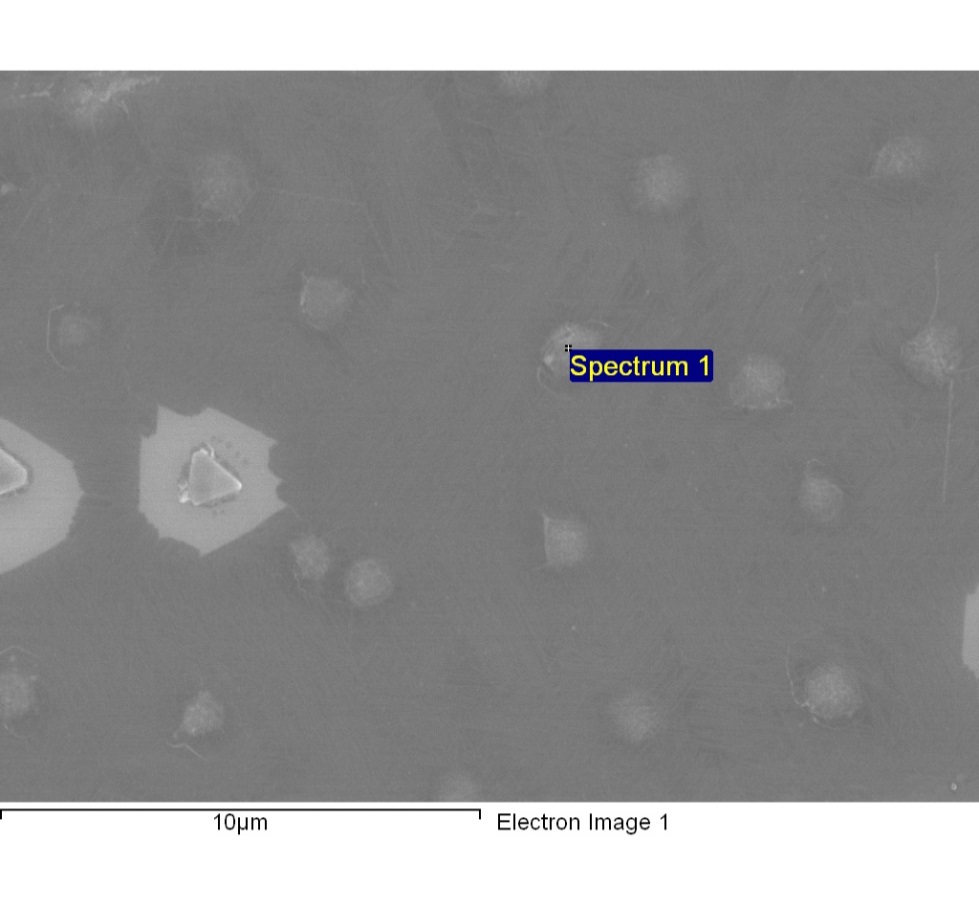

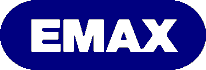


Spectrum processing :

Possible omitted peak : 1.044 keV

Processing option : All elements analyzed (Normalised)

Number of iterations = 4

Standard :

C CaCO3 1-Jun-1999 12:00 AM

O SiO2 1-Jun-1999 12:00 AM

Si SiO2 1-Jun-1999 12:00 AM

W W 1-Jun-1999 12:00 AM

| Element | Weight% | Atomic% |  |
| --- | --- | --- | --- |
|  |  |  |  |
| C K | 15.21 | 27.46 |  |
| O K | 20.49 | 27.78 |  |
| Si K | 56.82 | 43.88 |  |
| W M | 7.48 | 0.88 |  |
|  |  |  |  |
| Totals | 100.00 |  |  |

Comment:

S6


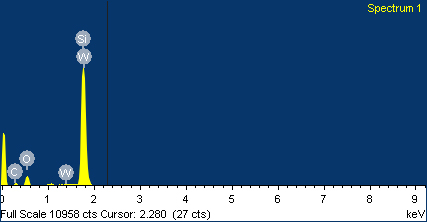


1. * Corresponding author, School of Science, China University of Geosciences, Beijing 100083, China

   Email address: xingjie@cugb.edu.cn [↑](#footnote-ref-1)
